# Feasibility of deuterium magnetic resonance spectroscopy of 3-O-Methylglucose at 7 Tesla

**DOI:** 10.1371/journal.pone.0252935

**Published:** 2021-06-07

**Authors:** Benedikt Hartmann, Max Müller, Lisa Seyler, Tobias Bäuerle, Tobias Wilferth, Nikolai Avdievitch, Loreen Ruhm, Anke Henning, Alexei Lesiv, Pavel Ivashkin, Michael Uder, Armin M. Nagel

**Affiliations:** 1 Institute of Radiology, University Hospital Erlangen, Friedrich-Alexander-Universität Erlangen-Nürnberg (FAU), Erlangen, Germany; 2 Max Planck Institute for Biological Cybernetics, Tübingen, Germany; 3 Advanced Imaging Research Center, UT Southwestern Medical Center, Dallas, Texas, United States of America; 4 Solvex Limited Liability Company, Moscow, Russia; 5 Division of Medical Physics in Radiology, German Cancer Research Center (DKFZ), Heidelberg, Germany; Henry Ford Health System, UNITED STATES

## Abstract

Deuterium Magnetic Resonance Spectroscopy (DMRS) is a non-invasive technique that allows the detection of deuterated compounds in vivo. DMRS has a large potential to analyze uptake, perfusion, washout or metabolism, since deuterium is a stable isotope and therefore does not decay during biologic processing of a deuterium labelled substance. Moreover, DMRS allows the distinction between different deuterated substances. In this work, we performed DMRS of deuterated 3-O-Methylglucose (OMG). OMG is a non-metabolizable glucose analog which is transported similar to D-glucose. DMRS of OMG was performed in phantom and in vivo measurements using a preclinical 7 Tesla MRI system. The chemical shift (3.51 ± 0.1 ppm) and relaxation times were determined. OMG was injected intravenously and spectra were acquired over a period of one hour to monitor the time evolution of the deuterium signal in tumor-bearing rats. The increase and washout of OMG could be observed. Three different exponential functions were compared in terms of how well they describe the OMG washout. A mono-exponential model with offset seems to describe the observed time course best with a time constant of 1910 ± 770 s and an offset of 2.5 ± 1.2 mmol/l (mean ± std, N = 3). Chemical shift imaging could be performed with a voxel size of 7.1 mm x 7.1 mm x 7.9 mm. The feasibility of DMRS with deuterium labelled OMG could be demonstrated. These data might serve as basis for future studies that aim to characterize glucose transport using DMRS.

## Introduction

Some diseases, such as cancer, are characterized by an increased glucose uptake [1]. A widely used technique for molecular imaging of glucose is positron emission tomography (PET). One of the most frequently used PET tracers is the radioactively labeled glucose analog 2-18F-fluoro-2-deoxy-D-glucose (FDG). After injection, FDG spreads in the body and accumulates in tissue with high glucose uptake [2]. To study glucose transport, also ^11^C labelled 3-O-Methylglucose (OMG) has been used [3]. As for all radioactive substances, special care is needed for the production, transport, and administration of ^18^F and ^11^C, which have a half-life time of 109.77 min and 20.3 min [4]. Here is where Deuterium Magnetic Resonance Spectroscopy (DMRS) and Imaging (DMRI) can intervene and provide a non-invasive alternative: Deuterium as a stable isotope is used instead of ^18^F or ^11^C and replaces protons of the glucose. The main principle during the measurement is similar: Tissue with a high glucose uptake like tumors and metastases accumulate the labelled substance. In contrast to PET, DMRS allows also the identification of different substances containing deuterium via chemical shift imaging (CSI). From the latter an analysis can be made of metabolic pathways and rates.

Beginning half a century ago [5, 6], deuterium has been used to replace protons in well-chosen substances, making it possible to quantify the concentrations, the processing, and the distribution of these substances in vivo. Some approaches and applications of DMRS and DMRI include measurements of blood flow and tissue perfusion [7], analysis of chronic graft-versus-host disease [8], and in vivo measurement of drug concentrations [9]. More recently, deuterated glucose has been used to investigate metabolism in rats and humans [10, 11]. Despite numerous benefits, some difficulties appear in DMRS: Compared to PET, DMRS has a much lower sensitivity and thus requires tracer concentrations at least in the range of mmol/l. Thus, glucose analogs seem to be interesting tracers since they can be injected in sufficiently high concentrations. Nevertheless, to obtain a good signal-to-noise ratio (SNR), ultra-high magnetic field MRI systems are needed [12] and voxel volumes have to be approximately two orders of magnitude larger than for ^1^H MRI. In addition, the short relaxation times of deuterium, which are caused by an electric quadrupole moment of the nucleus (deuterium has a nuclear spin of 1), require pulse sequences that allow short echo times. Another challenge for imaging without further enrichment is the low natural abundance of deuterium, which is 0.0156% [13]. Conversely, this is an advantage for studies with deuterium-labelled substances without the spectrum being dominated by naturally occurring deuterium.

In this work, we investigated deuterated OMG as potential tracer for DMRS. OMG is a non-metabolizable glucose analog. It is not accumulated in the body [14], in general considered non-toxic for diagnostic purposes [15], and therefore allows repetition of the experiment within a short period of time. OMG has been used to study transmembrane glucose transport in skeletal muscle using ^11^C as tracer [3], but with a half-life time of 20 minutes, ^11^C decays three times faster than ^18^F and causes a higher radiation exposure. It was recently used for chemical exchange saturation transfer (CEST) MRI on breast cancer models where it showed a stronger effect that also lasted longer than D-glucose (glucoCEST) [16]. OMG is therefore a promising candidate to examine glucose uptake, perfusion, washout or metabolism and oncological diseases.

In this work, chemical shift and relaxation times of OMG were determined in phantom measurements. To demonstrate the feasibility of DMRS with deuterated OMG at 7 Tesla, we used non-localized spectroscopy as well as CSI to monitor and to analyze the time course of the OMG concentration in a rat model of breast cancer bone metastases.

## Materials and methods

All measurements were performed on a horizontal 7 Tesla preclinical MR system (ClinScan 70/30, Bruker, Ettlingen, Germany). The MR system does not support the nucleus ^2^H by default. Instead, the nucleus ^17^O was selected in the scanner software because of its comparable gyromagnetic ratio (γ_17O_/γ_2H_ = 0.883) while the Larmor frequency of deuterium was provided externally with a signal generator. For that purpose, an E506B vector network analyzer (Agilent Technologies, Santa Clara, CA, USA) was used. A single resonant Tx/Rx body coil (Bruker, Ettlingen, Germany) for 300 MHz (^1^H) was used for positioning and shimming for in vivo measurements as well as phantom measurements together with a single resonant Tx/Rx loop coil (Stark Contrast, Erlangen, Germany) tuned to 46 MHz with a diameter of 2 cm for ^2^H data acquisition (a picture is given in S1 Fig). A double-resonant Tx/Rx birdcage coil for 300 MHz and 46 MHz with an inner diameter of 7 cm was used for further phantom measurements. All data sets were reconstructed offline with custom-written Matlab scripts (MATLAB, The MathWorks, Natick, MA, USA).

### Synthesis of 3-O-CD_3_-glucose (OMG)

OMG was obtained from Solvex, Russia, and produced as follows: NaH (60%) (1.1 g) is slowly added to a stirred solution of D-glucose diacetonide (Sigma-Aldrich, 5 g, 19.2 mmol) in DMF (10 mL) at 0°C. The reaction mixture is stirred for 1 hour at room temperature, and then CD_3_I (1.5 ml) is added dropwise. After 2 hours the reaction mixture is poured into ice water and the crude product is extracted with ethyl acetate. The organic phase is washed with water twice, then evaporated. The residue is dissolved in THF (15 mL); CF_3_COOH (2 mL) and water (1 mL) are added. The reaction mixture is heated to reflux for 5 hours. Then, 10 mL of toluene is added and the mixture is evaporated. 3-O-CD_3_-glucose (2.6 g, 70%) is purified by column chromatography on silica gel.

The purified 3-O-CD_3_-glucose has been repeatedly dissolved in water for injection and lyophilized to remove traces of organic solvents; finally filtered through a 0.2 μm sterile filter and lyophilized to produce a white powder.

The manufacturer investigated the degree of deuteration and the label loss. The deuteration was 3 deuterium atoms per OMG molecule and was calculated through comparison with commercially available deuterated substances. Furthermore, ^1^H and ^2^H NMR spectra were analyzed and showed no sign of protons in the methyl group. Potential label loss was investigated by analyzing solutions of OMG in water, PBS and saline at varying temperatures up to 100°C, again by ^1^H and ^2^H NMR spectroscopy. No loss has been observed, which indicates a strong binding of the deuterium to the methyl group of OMG.

### Animals

Male RNU rats were obtained from Charles River, Germany, and housed at the central animal facility of the University of Erlangen-Nuremberg. In total, eight animals were examined. All care and experimental procedures were performed in accordance with national and regional legislation on animal protection, and all animal procedures were approved by the State Government of Middle Franconia, Germany. At an age of six weeks, the animals underwent surgery to induce the metastases. For that purpose, the rats were inoculated with MDA-MB-231 breast cancer cells into the superficial epigastric artery of the right hind leg as described in [17].

During the DMRS experiments, all animals were anesthetized using isoflurane (2%, 2 L/min) applied through a nose cone. The animals were kept warm with a heated water pad and the respiratory rate was monitored with a pressure transducer.

### Relaxation times, chemical shift, and degree of deuteration of OMG

The relaxation times were measured with inversion recovery (IR) and spin echo (SE) sequences under fully relaxed conditions. The phantom has a cylindrical shape with a volume of 2 ml, a diameter of approximately 1 cm, and a length of approximately 3 cm. It contains a solution of 57.7 mg OMG in 2 ml distilled water, which results in a concentration of 149 mmol/l OMG. The loop coil was used for the measurements and placed on top of the phantom, whose orientation was parallel to the magnetic field. The parameters for the T_1_ measurement were as follows: Echo time (TE) = 0.35 ms (for free induction decay sequences, TE is defined as the period of time between the middle of the excitation pulse and the beginning of the readout), bandwidth 15 kHz, vector size 4096, radiofrequency (RF) pulses had a duration of 1000 μs and 500 μs, rectangular shape, and flip angles of 180° and 90°, respectively. To calibrate the flip angle, prior to each measurement, a global calibration using rectangular excitation pulses with variable amplitude was performed. A sinusoidal fit was used to determine the reference voltage (i.e. voltage required to achieve 180° flip angle for a rectangular pulse of 1 ms duration). Repetition time (TR) = (2.5 s + inversion time), and 17 different inversion times (TI) (10, 30, 80, 150, 250, 400, 600, 800, 1000, 1300, 1500, 1800, 2000, 2400, 2800, 3200, 3600 ms). For each TI, 25 averages were acquired. The parameters used for the T_2_ measurement were TR = (2.5 s + TE) with 14 echo times (15, 30, 60, 130, 170, 250, 350, 500, 800, 1100, 1300, 1600, 1800, 2000 ms). The bandwidth was 15 kHz, vector size 4096, 25 averages for each TE. The RF pulses had a rectangular shape, flip angles of 90° and 180°, and a duration of 500 μs and 1000 μs. The signal intensity for a measurement was determined by integration of the first 10 ms of the FID for each TI or TE value. For the T_1_ time, a two-parameter mono-exponential function of the form

$$\left|1+(M_{start}-1)\cdot exp(-(1/T_{1})\cdot t)\right|$$ (1)

was fitted to the data, where the parameter M_start_ takes into account the inversion efficiency. A three-parameter mono-exponential function of the form

$$A\cdot exp(-(1/T_{2})\cdot t)+\text{offset}$$ (2)

was fitted to the data to determine the T_2_ time. For the fit of the T_2_ time, the first echo was discarded.

The degree of deuteration and the chemical shift of OMG were measured with a spherical phantom containing a solution of 4.3 mmol/l OMG in distilled water using the water peak as internal reference. The water peak was set to 4.8 ppm. The ^1^H channel of the birdcage coil was used for positioning and shimming and the deuterium channel was used with a non-localized FID sequence for the acquisition of deuterium spectra with TR = 2 s, TE = 0.4 ms, 150 averages and a bandwidth of 2 kHz. The RF pulse was a 90° pulse with rectangular shape and a duration of 620 μs. 2048 data points were acquired, zero-filled to 22528 data points and then transformed via Fourier transform to obtain the spectrum. The spectrum was fitted with a bi-Lorentzian function of the form

$$L_{2}(f)=A_{1}/\left({\left(f-f_{0,1}\right)}^{2}+0.25\Gamma_{1}^{2}\right)+A_{2}/\left({\left(f-f_{0,2}\right)}^{2}+0.25\Gamma_{2}^{2}\right)+\text{offset}$$ (3)

to determine the peak area and the peak positions. A_1_ and A_2_ are the amplitudes, f_0,1_ and f_0,2_ the positions of the maxima and Γ_1_ and Γ_2_ denote the full width at half maximum (FWHM) of the peaks. 5000 data points at each end of the spectrum (corresponding to parts of the spectrum that are more than 560 Hz away from the water peak) were neglected for the fitting.

### Deuterium MRS

Animal positioning and B_0_-shimming were performed using the dedicated ^1^H body coil (Bruker), the mean linewidth of the water peak was 224 ± 33 Hz. Each rat was positioned head first-prone, with the loop coil positioned next to the tumor-bearing leg. The knee of the leg served as a reference point to center the coil over the tumor. A T_2_ weighted turbo spin echo (TSE) sequence was used to acquire morphological images. 40 slices with a field of view of 65 mm x 55 mm, matrix 320 x 272, and a thickness of 1 mm were acquired with TR = 5800 ms, TE = 36 ms, and flip angle 180°. After that, the network analyzer was connected to the technical cabinet of the scanner for deuterium measurements. All spectra were acquired using a non-localized FID sequence and the ^2^H loop coil with the following parameters: TR = 250 ms, TE = 0.35 ms, flip angle 68° (Ernst-angle for an assumed in vivo T_1_ of 250 ms), rectangular pulse with duration 500 μs. A relatively high bandwidth of 178,6 kHz (4096 complex data points) was chosen, due to the short in vivo transverse relaxation times. 14000 averages were acquired in total without interruption or delay. Deuterated OMG was injected intravenously via catheter into the tail vein 200 seconds after the beginning of data acquisition and over a period of 55 seconds. An injection contained a solution of 0.89 g OMG per kg body weight. The final data were processed as follows: The FID time domain data were zero-filled to 65536 data points, divided into packs of 20 averages each and transformed into the frequency domain via Fourier transform. All peaks in the deuterium spectra were then fitted with a Lorentz profile using a custom-written Matlab script. The pre-injection spectra were fitted by using a mono-Lorentz profile of the form

$$L_{1}(f)=A/\left({\left(f-f_{0}\right)}^{2}+0.25\cdot \Gamma^{2}\right)+\text{offset}$$ (4)

with amplitude *A*, FWHM Γ and position of the maximum *f*_0_, followed by a calculation of the peak area since there is only the water peak. For the time after the injection, the mono-Lorentzian function given in Eq 4 was used to determine the peak areas as follows: first, the baseline spectrum (averaged over all pre-injection spectra) was subtracted and the OMG peak fitted. After that, the OMG fit was subtracted from the spectrum and the water peak was fitted. The signal amplitudes were then normalized to the baseline water signal and the deuterium concentrations were calculated corresponding to a baseline deuterium concentration of 13 mmol/l. This concentration was derived from an assumed water content fraction of 75% in muscle tissue [18] and the natural abundance of 0.0156% [13]. The concentration of deuterium in water and in OMG was plotted over time and three exponential functions were fitted to the OMG data to describe the washout, taking into account the data from the maximum of the OMG curve until its end: A mono-exponential function without offset

A⋅exp(-(1/α)⋅t) (5)

a mono-exponential function with offset

A⋅exp(-(1/α)⋅t)+offset (6)

and a bi-exponential function without offset

A⋅exp(-(1/α)⋅t)+B⋅exp(-(1/β)⋅t) (7)

These functions were chosen because of the shape of the curve and previous observations by McWhorter et al. [19] and Chang et al. [20].

FID measurements were followed by 2D-CSI with the following parameters: Slice selective excitation (Sinc pulse, duration = 1.28 ms, time-bandwidth-product = 3.55, nominal slice thickness = 7.9 mm), TR = 250 ms, TE = 2.3 ms, flip angle 68° (Ernst-angle for an assumed in vivo T_1_ of 250 ms), 250 averages, bandwidth 90 kHz, 4096 complex data points. A grid of 4 x 5 voxels, each with nominal resolution 7.1 x 7.1 x 7.9 mm^3^, was encoded, resulting in a total FOV of 28.3 mm x 35.3 mm. The CSI data were reconstructed via two dimensional Fourier transform for the spatial dimensions. The time domain data was zero-filled to 22528 data points and converted to the frequency domain via 1-D Fourier transform.

## Results

### Relaxation times, chemical shift, and degree of deuteration of OMG

**Fig 1:**
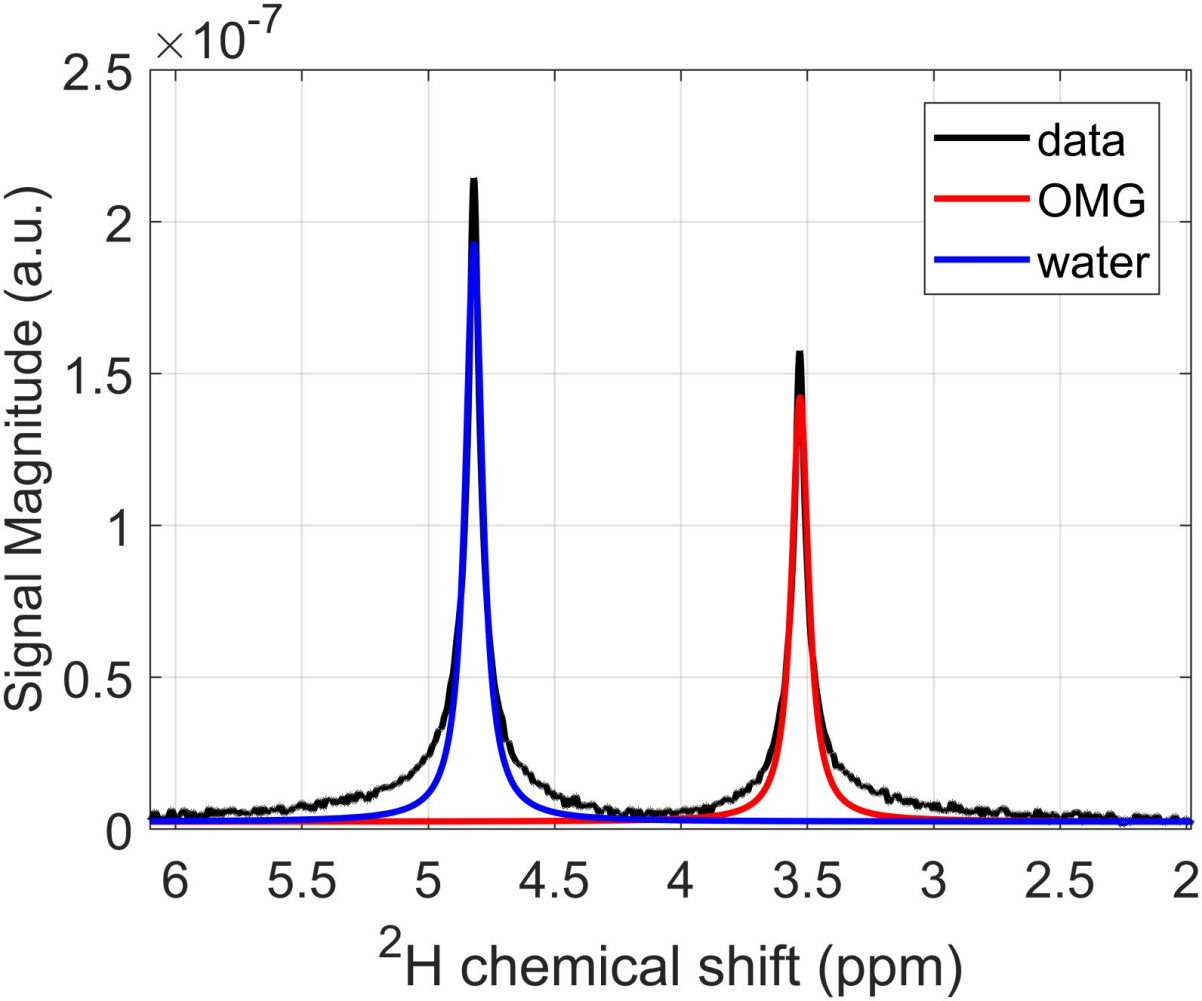
Deuterium spectrum of the OMG phantom. Deuterium spectrum of the spherical phantom containing 4.3 mmol/l OMG and distilled water. We assume a natural abundance of deuterium of 0.0156%. The chemical shift was determined to 3.51 ± 0.01 ppm. From a comparison of the peak areas, a degree of deuteration of 2.9 ± 0.1 deuterium atoms per OMG molecule could be estimated.

The relaxation times of deuterium in OMG in a solution with water are T_1_ = 452 ± 2 ms and T_2_ = 406 ± 21 ms. The chemical shift is 3.51 ± 0.01 ppm, see Fig 1. From the comparison of the OMG peak area with the water peak area, a degree of deuteration of 2.9 ± 0.1 deuterium atoms per OMG molecule could be determined.

### Deuterium MRS

**Fig 2:**
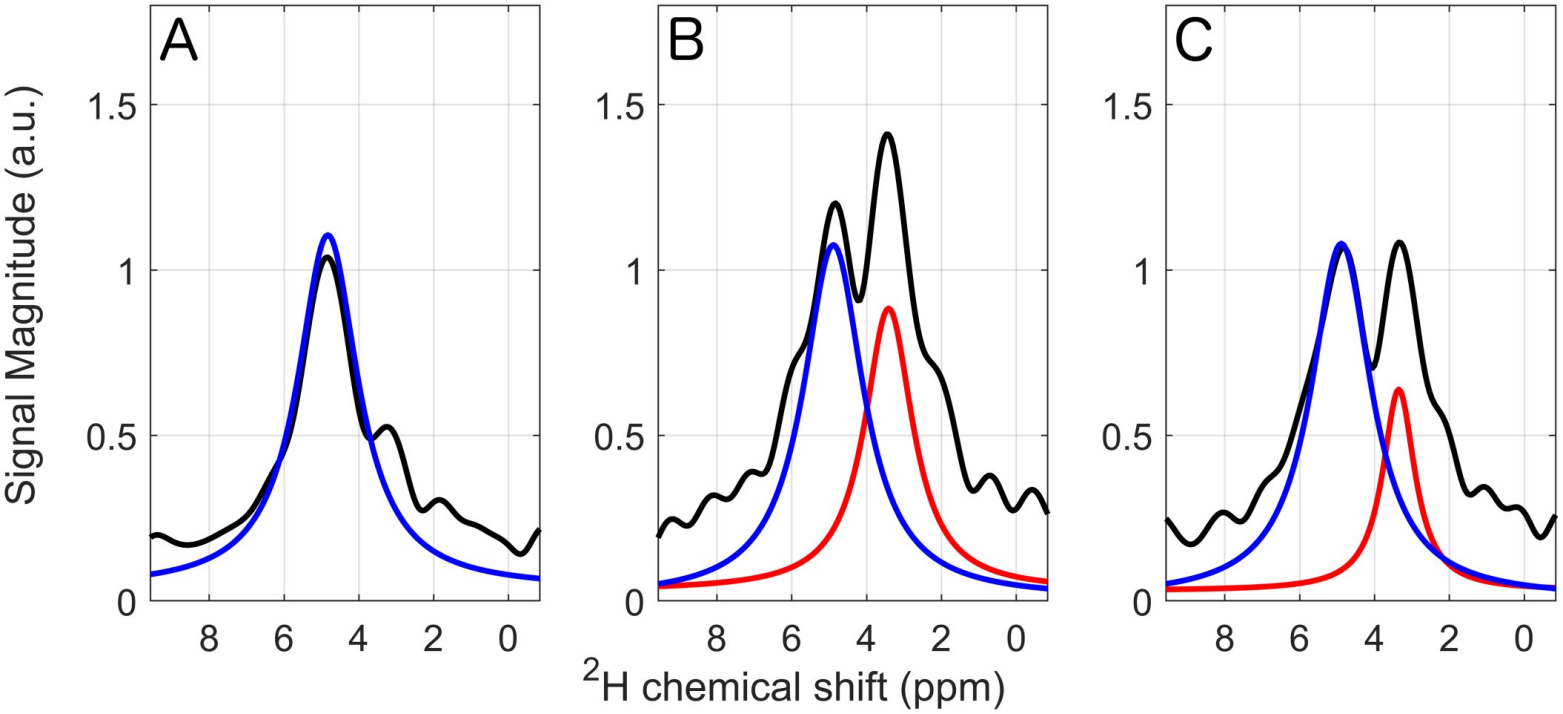
Deuterium spectra of rat 3 at different time points. Mono-Lorentzian functions were used to separately fit the different peaks. The water peak serves as internal reference and was set to 4.8 ppm. **A** Before the injection. The natural abundant deuterium in water is visible. **B** 170 s after the injection. The water and OMG signals are visible. **C** 55 minutes after the injection. Both peaks are visible, but the OMG signal shows a decreased amplitude.

Three of the deuterium spectra acquired in vivo in rat three are shown in Fig 2. After shimming, the deuterium peak of water (HDO) had a mean linewidth of 110 ± 14 Hz. For each spectrum, 20 data sets were averaged. Spectrum A represents the time point at t = 195 s immediately before the injection and shows only one peak, the natural abundance of deuterium in water (set to 4.8 ppm). Spectrum B was recorded at t = 370 s, about 115 s after the end of the injection. It shows the additional glucose signal at approximately 3.5 ppm. Spectrum C marks the end of the experiment, it was recorded 3240 s after the injection at t = 3495 s. The magnitude of the glucose signal has already decreased compared to spectrum B. Other peaks are not visible.

**Fig 3:**
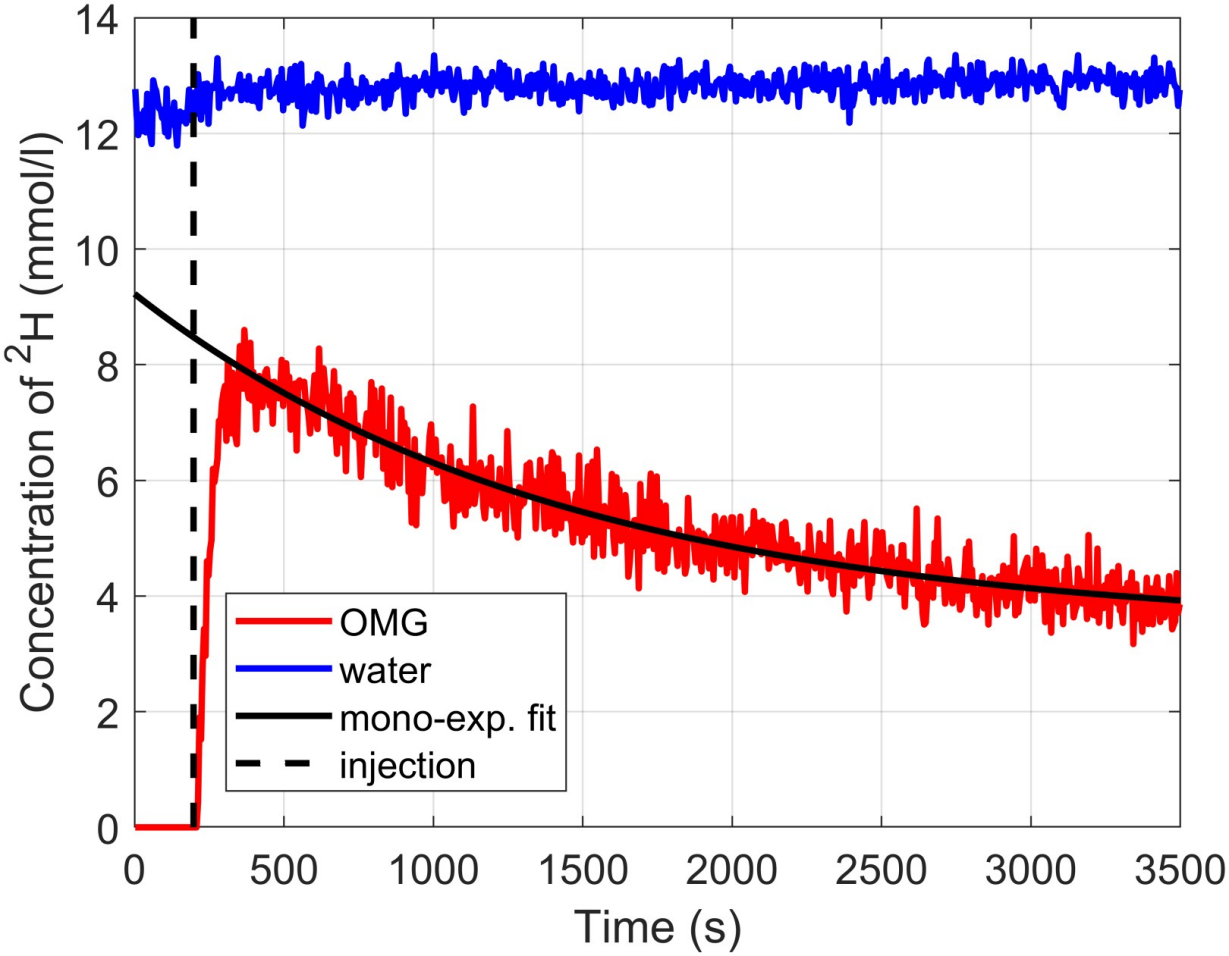
Time evolution of the signal amplitudes in the deuterium spectrum. Signal amplitudes of the different peaks in the deuterium spectrum are shown for the whole time course of the experiment (result from rat 3). The concentration of ^2^H within both chemical environments (water and OMG) is given. The concentration of OMG itself can be obtained by dividing the data by its degree of deuteration, which is 2.9. The injection of OMG took place at t = 200 s and was followed by a steep increase of the glucose signal. The washout of OMG was fitted with a mono-exponential function with offset, taking into account the data from its maximum (at t = 368 s) until the end.

Fig 3 shows the time course of the concentration of deuterium in water and OMG. The concentration of deuterium in OMG rises steeply after the injection. It increases a little bit slower before it reaches its absolute maximum at t = 368 s. At the end of the experiment, the concentration reached a level of approximately 4 mmol/l. The results of the fitted washout functions are given in Table 1. The mono-exponential model with offset seems to fit the washout curve best, while the fitting of the bi-exponential model was not stable as indicated by the large uncertainties output by the fitting software. The water peak shows a slight increase after the injection from approximately 12.3 mmol/l to approximately 12.8 mmol/l and then stays constant over the time course of the experiment. Summarizing the observations of all three rats, it can be stated that the glucose peak rises steeply after the infusion, reaches a maximum approximately 500 s (mean over all three animals = 496 s) after the infusion, and then decreases. One hour after the injection, the glucose peak has dropped to about 40% of its maximum value.

**Table 1:** Washout rates of OMG in vivo.

		Rat 1	Rat 2	Rat 3	Mean ± Std
Mono-exp. Model	A (mmol/l)	9.4 ± 0.2	9.9 ± 0.1	8.1 ± 0.1	9.1 ± 0.2
	α (s)	3340 ± 110	3470 ± 90	4250 ± 140	3690 ± 500
	R^2^	0.8532	0.9198	0.8599	
Mono-exp. Model with offset	A (mmol/l)	7.6 ± 0.2	9.1 ± 0.6	5.8 ± 0.2	7.5 ± 1.7
	α (s)	1510 ± 190	2790 ± 520	1430 ± 160	1910 ± 770
	Offset (mmol/l)	3.0 ± 0.3	1.1 ± 0.8	3.4 ± 0.2	2.5 ± 1.2
	R^2^	0.8702	0.9205	0.8871	
Bi-exp. Model	A (mmol/l)	7.5 ± 5.0	9.1 ± 27.9	4.1 ± 1.4	6.9 ± 2.6
	α (s)	1480 ± 1060	2800 ± 6380	910 ± 490	1720 ± 960
	B (mmol/l)	3.1 ± 5.4	1.1 ± 28.5	5.4 ± 1.8	3.2 ± 2.2
	β (s)	107770 ± 4122740	143600 ± 67512900	9580 ± 7610	87000 ± 69400
	R^2^	0.8702	0.9205	0.8876	

Results of fits of deuterium concentration in OMG versus time from experiments with three rats. All errors represent the confidence intervals given by the fitting software.

**Fig 4:**
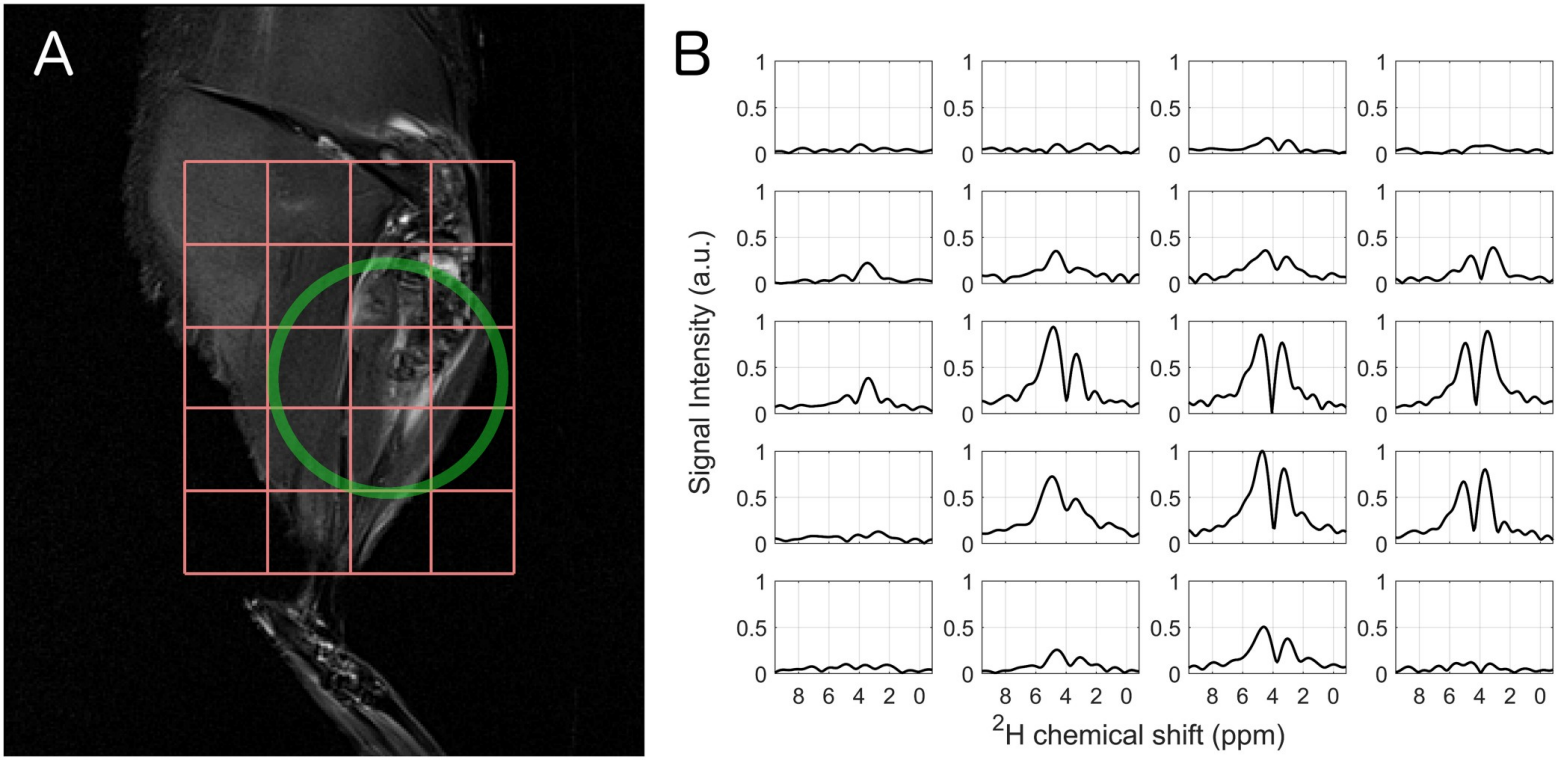
Chemical shift imaging of the rat leg (results from rat 3). **A** Position of the grid relative to the leg and approximate position of the coil. The image was acquired with a T_2_ weighted turbo spin echo sequence and shows the metastases in the upper right half of the grid. The image represents a 1 mm thick slice within the CSI volume. **B** The deuterium spectra show an inhomogeneous intensity distribution due to the sensitivity profile of the loop coil, yet the OMG and water peak are clearly visible and distinguishable.

In Fig 4A, the position of the CSI grid relative to the rat leg with the corresponding spectra (Fig 4B (data of rat 3)) is visualized. Not all voxels show a signal, with almost no signal at all in the top row. In all spectra that do show a signal, the two peaks of water and OMG are distinguishable. While the voxels in the middle of the region of interest (second and third column, second to fourth row) show a stronger water than OMG peak, the voxels in both outer columns show a stronger OMG peak.

## Discussion

In this work, the feasibility of DMRS using deuterated OMG was demonstrated in phantom and in vivo measurements using a rat model of breast cancer bone metastasis. In previous work, deuterated D-glucose was used for DMRS, which allows also the analysis of metabolites such as lactate and glutamate [11]. In contrast, OMG is non-metabolizable and, thus, was previously used as PET tracer to study glucose transport [3]. We could show that deuterated OMG has good properties for DMRS. The chemical shift is 30% larger than for the signal of D-Glucose-6,6-d_2_, which was reported in recent publications in this field [10, 11], making it a little bit easier to distinguish the peaks in the spectrum. OMG has little or no effect on the hormone regulation of the blood sugar level, thus allowing much higher concentrations of this sugar in the blood than for D-glucose [21]. In the future, this might also allow to increase the SNR.

### Relaxation times, chemical shift, and degree of deuteration of OMG

At 7 Tesla, only little information about relaxation times of deuterium is available so far (see S1 Table). Therefore, to compare the relaxation times of OMG, we measured relaxation times of natural abundant deuterium in water (HDO) in phantoms as well as in vivo (see S1 File and S2 and S3 Figs). The relaxation times of deuterium in OMG in a solution with distilled water are similar, yet a little smaller than for deuterium in water itself. The use of a loop RF coil in combination with non-adiabatic pulses results in non-uniform flip angle distribution, which might reduce the accuracy of the measurements. This should be refined in further work by employing volumetric RF coils with a homogenous B_1_ profile together or with adiabatic pulses, tailored specifically for this application. The degree of deuteration was determined using the water peak as internal reference with a deuterium concentration of 17.3 mmol/l and yielded 2.9 ± 0.1 deuterium atoms per OMG molecule, which agrees well to the expected value of three. Using the natural abundance as internal reference is a compelling way to quantify concentrations. However, there is an uncertainty in the assumed natural abundance since it shows a broad variation depending on the origin of the sample [22]. For future measurements, well-calibrated external references might be used to improve the accuracy.

### Deuterium MRS

OMG is transported via glucose transporters into the cell but is also permeable to the cell membrane, such that it equilibrates between the cell and the extracellular space [23]. The time course of the OMG concentration can be monitored with a high temporal resolution of 5 s (20 averages with TR = 250 ms). The washout of OMG can be well described with a mono-exponential function with offset. According to Csaky [14] and Campbell [21], OMG is washed out of the organism to a high degree via renal filtration. According to Chang et al. [20] and McWhorter et al. [19], the washout shows a bi-exponential behavior. McWhorter measured washout time constants of 1300 s and 7100 s for fast and slow component, respectively. Chang et al. observed washout time constants of 1800 s and 7900 s. The results of rat 3 show similar numbers, yet the first two rats yield much longer time constants for the slowly decreasing component. Although a mono-exponential model with offset gives the best fit results, it remains unclear why there should be an offset. If OMG is filtered out by the kidney as observed by Campbell [21] and Csaky [14], one would expect that the kidneys will filter out all of the OMG. Since the duration of our experiment was about an hour, it is very likely that the data acquisition was not long enough to capture enough data points to observe a bi-exponential washout.

Interestingly, Hwang et al. [24] observed an increasing OMG concentration in murine RIF-1 tumors over a time of 2 hours before reaching a plateau. This indicates that the tumors absorb and process OMG differently, which might be relevant for oncological diagnosis.

The deuterium spectrum in Fig 2A shows a relatively broad linewidth (110 Hz / 46 MHz = 2.3 ppm) compared to the ^1^H spectrum (224 Hz / 300 MHz = 0.75 ppm), which is probably caused by a shorter T_2_ time of deuterium and larger B_0_ inhomogeneities within the small FOV of the ^2^H loop coil. In addition, it shows an asymmetry of the water peak, indicating that there are other potential contributions than water or B_0_ inhomogeneities. Further analysis about the origin of this peak is needed in future work to increase the precision of the quantification of deuterated compounds. Furthermore, the bi-Lorentzian fitting is not trivial, given the not purely Lorentzian shape of the peaks and the noise or background of small unidentified peaks. The fitting was stable with respect to varying the start parameters. However, the overlap of the peaks might reduce the accuracy of the quantification. Nonetheless, a possible bias should be approximately constant for all spectra und only pose a minor influence on the washout model. Another factor that might affect the quantification of the OMG concentration is a potential saturation effect due to different T_1_ times of HDO and OMG. Here, we assume similar T_1_ times, as observed in the phantom measurements, yet the relaxation times of OMG in vivo should be quantified for further studies in order to increase precision.

The concentration of deuterium in water rises promptly after the infusion, it does not need as much time as the glucose to reach a maximum. It is possible that the water fraction in the tissue of interest increased and that there is therefore more deuterium that can be detected. A rise of the water peak due to newly produced water containing deuterium from the injected OMG as a byproduct of glycolysis, as mentioned by Lu [10], can be excluded, since the OMG is not metabolized [21] and even if it was, the water signal would rise slower and not with a jump. OMG being non-metabolizable is also the reason why no other metabolites could be observed.

Although OMG could be detected in tumor tissue, it is not clear how much of the OMG was taken up by the muscle, making it difficult to determine the contributions of the different tissue types. The general feasibility of OMG CSI could be demonstrated with sufficient spectral resolution and SNR. However, despite sufficient SNR, a detailed comparison to healthy tissue could not be performed since the sensitivity profile of the RF coil used was not large enough to evenly cover a sufficient number of voxels containing both types of tissue. Furthermore, the loop coil exhibits an inhomogeneous transmit profile. In the future, a transmit birdcage coil should be combined with a receive surface coil array to achieve homogenous excitation as well as sufficient SNR and coverage. Concerning the sequence side, the 2D-CSI sequence was used in this work as provided by the manufacturer without modification of the excitation pulses or the readout. The relatively short excitation time in combination with the relatively low time-bandwidth product possibly produced a rather soft excitation profile. In future work, the sequence should be modified and tailored specifically to the application. The excitation duration should be increased in a reasonable manor, while also accounting for the fast relaxation times. Time-bandwidth product should be increased within the possible range to produce a cleaner slice profile with lower transition width. Furthermore, if the duration of the CSI sequence can be increased with respect to the total available measurement time, TR should be increased (and the corresponding Ernst angle adapted accordingly). This would give a better range of T_1_ for which near optimal SNR is achieved.

## Conclusion

At 7 Tesla, we investigated the feasibility of DMRS and CSI using deuterated OMG. In this work, it was shown that DMRS can be used to monitor the washout of deuterium-labelled OMG with high temporal resolution of 5 s in vivo, allowing to observe dynamical changes and not only steady state. Furthermore, the relaxation times of OMG and its chemical shift were measured. Given the promising results from this work and recent publications [10, 11, 25], DMRS and DMRI might complement PET examinations by allowing a gentler diagnosis without the need of ionizing radiation.

## Supporting information

S1 File Supporting material. (DOCX)

S1 Fig Loop coil. Tx/Rx loop coil for 46 MHz (deuterium). The diameter is 2 cm.
(TIF)

S2 Fig Longitudinal relaxation in vivo. Longitudinal relaxation time of natural abundant deuterium in water (HDO) T_1_ = 248 ± 7 ms (mean and standard deviation), N = 4.
(TIF)

S3 Fig Transverse relaxation in vivo. Transverse relaxation time of natural abundant deuterium in water (HDO) T_2_ = 11.4 ± 1.3 ms (mean and standard deviation), N = 4.
(TIF)

S1 Table Overview of relaxation times of deuterium. All values are given as mean ± standard deviation. IR = inversion recovery, SE = spin echo, Glx = glutamate + glutamine; n. a. = not available, Water = HDO.
(DOCX)

## Abbreviations

CEST: chemical exchange saturation transfer
CSI: chemical shift imaging
DMRI: deuterium magnetic resonance imaging
DMRS: deuterium magnetic resonance spectroscopy
FDG: 2-18F-fluoro-2-deoxy-D-glucose
FID: free induction decay
FOV: field of view
FWHM: full width at half maximum
IR: inversion recovery
OMG: 3-O-Methylglucose
PET: positron emission tomography
SE: spin echo
SNR: signal-to-noise ratio
TI: inversion time
TSE: turbo spin echo
