# Supplementary material for: Feasibility of deuterium magnetic resonance spectroscopy of 3-O-Methylglucose at 7 Tesla
Source: PLoS One. 2021 Jun 7;16(6):e0252935. doi: 10.1371/journal.pone.0252935 (PMC8184010; doi:10.1371/journal.pone.0252935)
Supplement: S1 File — (DOCX) [file pone.0252935.s001.docx]

**Feasibility of deuterium magnetic resonance spectroscopy of 3-O-Methylglucose at 7 Tesla**

Benedikt Hartmann, Max Müller, Lisa Seyler, Tobias Bäuerle, Tobias Wilferth, Nikolai Avdievitch, Loreen Ruhm, Anke Henning, Alexei Lesiv, Pavel Ivashkin, Michael Uder, Armin M. Nagel

Supporting Methods

**Relaxation times in water, agarose gel, and heavy water**

A spherical phantom was filled with distilled water and a small amount of heavy water (99.9 Atom%D, Carl Roth, Karlsruhe, Germany) such that the total deuterium concentration was 1 mol/l. This concentration was used to achieve higher SNR. The relaxation times of natural abundant deuterium in water (HDO) were measured in another phantom with agarose gel. Agarose is often used to mimic texture and relaxation times of muscle tissue. This phantom contains water, 5 % agarose gel (Carl Roth, Karlsruhe, Germany), and 20 mmol/l sodium chloride. Measurements on both phantoms were conducted with the ^2^H channel of the birdcage coil using inversion recovery (IR) and spin echo (SE) sequences under fully relaxed conditions. For the phantom containing deuterium-enriched water, the T_1_ time was determined using an echo time (TE) of 0.5 ms, a repetition time of TR = (2.5 s + inversion time (TI)) with 18 TI values (10, 50, 120, 200, 350, 450, 580, 700, 850, 1000, 1200, 1500, 1800, 2000, 2400, 2800, 3300, 3800 ms). Both RF pulses had a rectangular shape, the 180° pulse had a duration of 1360 µs, the 90° pulse of 680 µs. 4096 complex data points were acquired with a bandwidth of 10 kHz and 50 averages for each TI. The T_2_ measurements were performed with TR = (2.5 s + TE), TE = (15, 30, 60, 130, 170, 250, 350, 500, 800, 1100, 1400, 1600, 1800, 2000) ms, bandwidth 15 kHz, vector size 4096 and 30 averages. Both RF pulses had a rectangular shape, the 180° pulse had a duration of 1520 µs and the 90° pulse of 760 µs. For the agarose phantom, 16 different TI (10, 20, 40, 60, 80, 110, 140, 180, 240, 300, 400, 600, 800, 1000, 1300, 1500 ms) and 11 different TE (3, 5, 7, 10, 12, 15, 17, 19, 21, 23, 30 ms) were measured with 150 averages each. For both, T_1_ and T_2_ measurements, the bandwidth was set to 161.3 kHz, the vector size was 4096, and both RF pulses in each sequence had a rectangular shape, the 180° pulse had a duration of 1400 µs and the 90° pulse of 700 µs. The signal intensity for a measurement was determined by integration of the first 10 ms of the FID for each TI or TE value. T_1_ and T_2_ were fitted using the formulas in Equations 1 and 2. Pure heavy water (99.9 Atom%D, Carl Roth, Karlsruhe, Germany) was measured in a spherical phantom and the ^2^H channel of the birdcage coil. Both, the IR sequence and the SE sequence were used under fully relaxed conditions with TR = (2.5 s + TI) and TR = (2.5 s + TE), respectively. Both RF pulses in each sequence had a rectangular shape, the 180° pulse had a duration of 1640 µs, the 90° pulse of 820 µs. The echo time was set to 0.6 ms for the IR experiment, with 23 different TI times (15, 40, 100, 150, 200, 250, 300, 400, 500, 600, 700, 800, 900, 1000, 1150, 1300, 1450, 1600, 1800, 2000, 2400, 2800, 3200 ms). 15 different echo times were measured (20, 40, 70, 100, 150, 200, 300, 400, 500, 700, 900, 1100, 1300, 1500, 1900 ms). For each data point, 40 averages of an FID with 4096 complex data points were measured with a bandwidth of 10 kHz. The T_1_ and T_2_ times were obtained as before by integration of the first 10 ms of each FID and fitting of the Equations 1 and 2, respectively. All measurements were performed at room temperature.

**Relaxation times in vivo**

The relaxation times of natural abundant deuterium of water (HDO) were measured in vivo in rat muscle tissue. The loop coil was placed on the thigh muscle. Four rats were used for T_1_ and T_2_ measurements each, using the IR and the SE sequences under fully relaxed conditions. For the IR experiment, eleven inversion times (15, 40, 90, 150, 200, 300, 400, 500, 650, 1000, 1200 ms) were used. 190 averages were acquired for each inversion time with TR = (1.3 s + TI), TE = 0.35 ms, 4096 data points, and a bandwidth of 178.6 kHz. Both RF pulses had a rectangular shape, the 180° pulse had a duration of 1000 µs and the 90° pulse of 500 µs. For the SE measurement, eleven different echo times (5, 8, 11, 13, 15, 19, 23, 28, 33, 37, 45 ms) were measured with 180 averages each, TR = (1.3 s + TE), vector size 4096, and bandwidth 178.6 kHz. Both RF pulses had a rectangular shape, the 180° pulse had a duration of 1000 µs and the 90° pulse of 500 µs. The FID data of each single measurement was zero-filled to 45056 data points and Fourier transformed into frequency domain. The signal intensity for a measurement was determined by fitting a mono-Lorentz profile as given in Equation 4 to the spectrum and calculation of the peak area. The T_1_ and T_2_ values were then determined by fitting of Equations 1 and 2 to these signal intensities. The T_2_* relaxation time was determined from the FWHM of the Lorentzian fit from separately acquired deuterium spectra (TR = 1300 ms, TE = 0.35 ms, 180 averages, bandwidth 178.6 kHz, vector size 4096, zero-filling to 65536) as 1/(πFWHM).

**Supporting Results**

**Relaxation times in water, agarose, and heavy water**

The relaxation times of deuterium in water (HDO) are T_1_ = 470 ± 1 ms and T_2_ = 458 ± 5 ms. The measurements on the agarose phantom with natural abundant deuterium yielded T_1_ and T_2_ values of 270 ± 7 ms and 10.8 ± 0.4 ms, respectively. In pure heavy water, the relaxation times are T_1_ = 392 ± 1 ms and T_2_ = 375 ± 9 ms.

**Relaxation times in vivo**

The T_1_ measurements of natural abundant deuterium (HDO) in vivo yielded a relaxation time of 248 ± 7 ms and the T_2_ value was determined to be 11.4 ± 1.3 ms (both times: mean and standard deviation, N = 4). The data and fits are shown in S2 and S3 Figs in the supplementary material. From the FWHM of the water peak, T_2_* = 2.7 ± 0.1 ms (mean and standard deviation, N = 8) could be determined.

**Supporting Discussion**

**Relaxation times in water, agarose, heavy water, and in vivo**

The relaxation times measured in this work are in good agreement to the reported values in the literature, see S2 Table. For water, Borle [1] and Cope [2] report values similar to our results, so that we can confirm T_1_ times of approximately 450 ms for HDO at room temperature. The relaxation times measured in the 5 % agarose gel could not be compared to literature values since there are no values available, yet it is known that agarose significantly reduces T_2_ times compared to liquid water [3], which can be seen in our data. The relaxation times are similar to the in vivo results, indicating that such a phantom is well suited to mimic muscle tissue. The relaxation of pure D_2_O fits well to the results of Cope [2] and Woessner [4] and seem not or only slightly dependent on the magnetic field strength.

The relaxation in vivo is clearly faster than in phantoms. The T_1_ time is about half as short and the T_2_ time is approximately 40 times shorter than in the phantom. There have been in vivo measurements at a similar field strength with comparable results, for example Ackerman [5] measured T_1_ = 250 ms in vivo at 8.5 T and Assaf [6] measured T_2_ = 33 ms in vivo at 8.45 T.

**Supporting References**

1. Borle F, Seelig J. Hydration of Escherichia coli lipids. Deuterium T1 relaxation time studies of phosphatidylglycerol, phosphatidylethanolamine and phosphatidylcholine. Biochim Biophys Acta. 1983;735(1):131-6.

2. Cope FW. Nuclear Magnetic Resonance Evidence using D2O for Structured Water in Muscle and Brain. Biophysical Journal. 1969;9(3):303-19.

3. Mitchell MD, Kundel HL, Axel L, Joseph PM. Agarose as a tissue equivalent phantom material for NMR imaging. Magnetic Resonance Imaging. 1986;4(3):263-6.

4. Woessner DE. Molecular Reorientation in Liquids. Deuteron Quadrupole Relaxation in Liquid Deuterium Oxide and Perdeuterobenzene. The Journal of Chemical Physics. 1964;40(8):2341-8.

5. Ackerman JJ, Ewy CS, Becker NN, Shalwitz RA. Deuterium nuclear magnetic resonance measurements of blood flow and tissue perfusion employing 2H2O as a freely diffusible tracer. Proc Natl Acad Sci U S A. 1987;84(12):4099-102.

6. Assaf Y, Navon G, Cohen Y. In vivo observation of anisotropic motion of brain water using 2H double quantum filtered NMR spectroscopy. Magn Reson Med. 1997;37(2):197-203.
