# Supplementary figures and images for: Feasibility of deuterium magnetic resonance spectroscopy of 3-O-Methylglucose at 7 Tesla
Source: PLoS One. 2021 Jun 7;16(6):e0252935. doi: 10.1371/journal.pone.0252935 (PMC8184010; doi:10.1371/journal.pone.0252935)

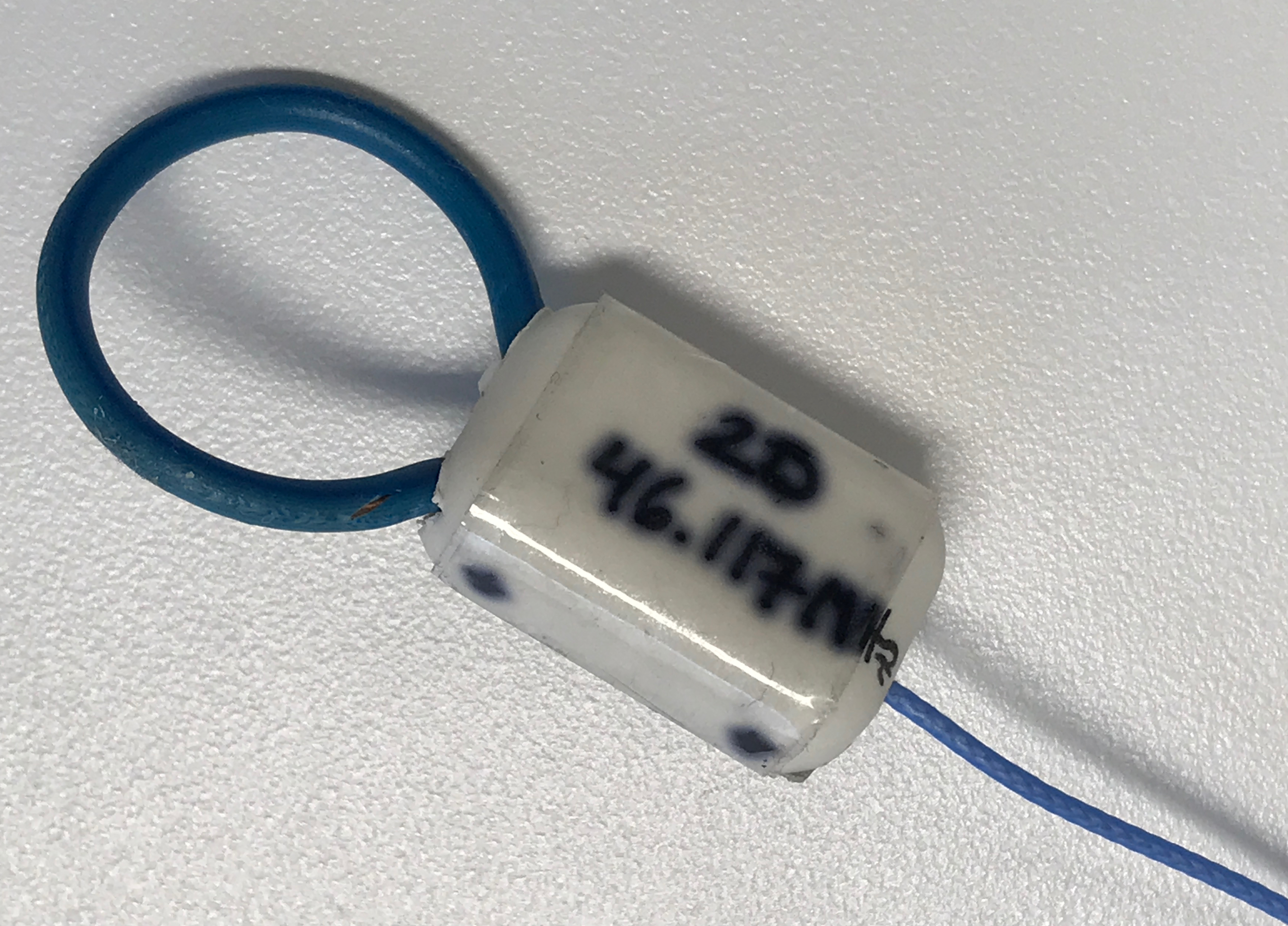

Supplement: S1 Fig — Tx/Rx loop coil for 46 MHz (deuterium). The diameter is 2 cm. (TIF) [file pone.0252935.s002.tif]

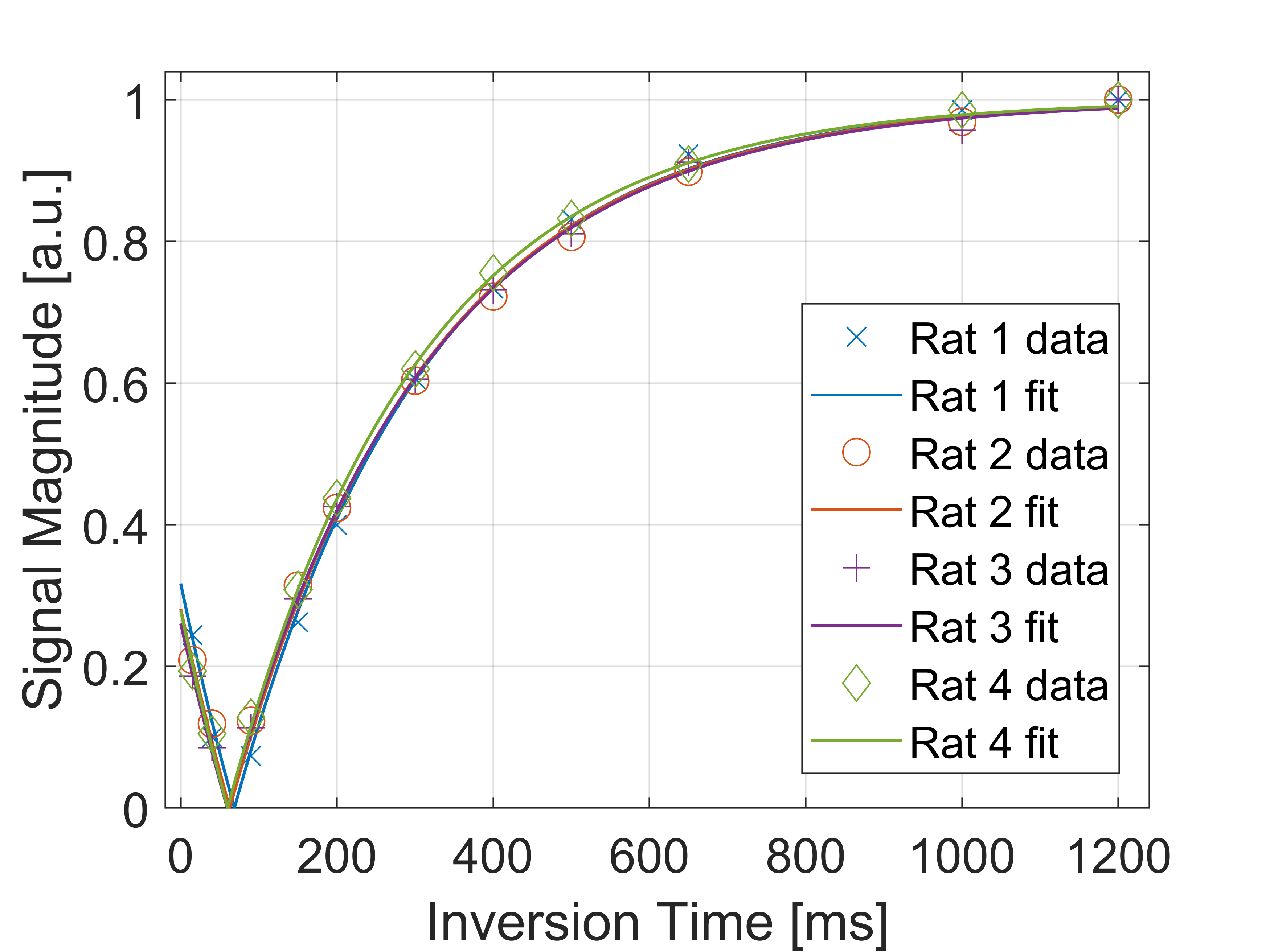

Supplement: S2 Fig — Longitudinal relaxation time of natural abundant deuterium in water (HDO) T1 = 248 ± 7 ms (mean and standard deviation), N = 4. (TIF) [file pone.0252935.s003.tif]

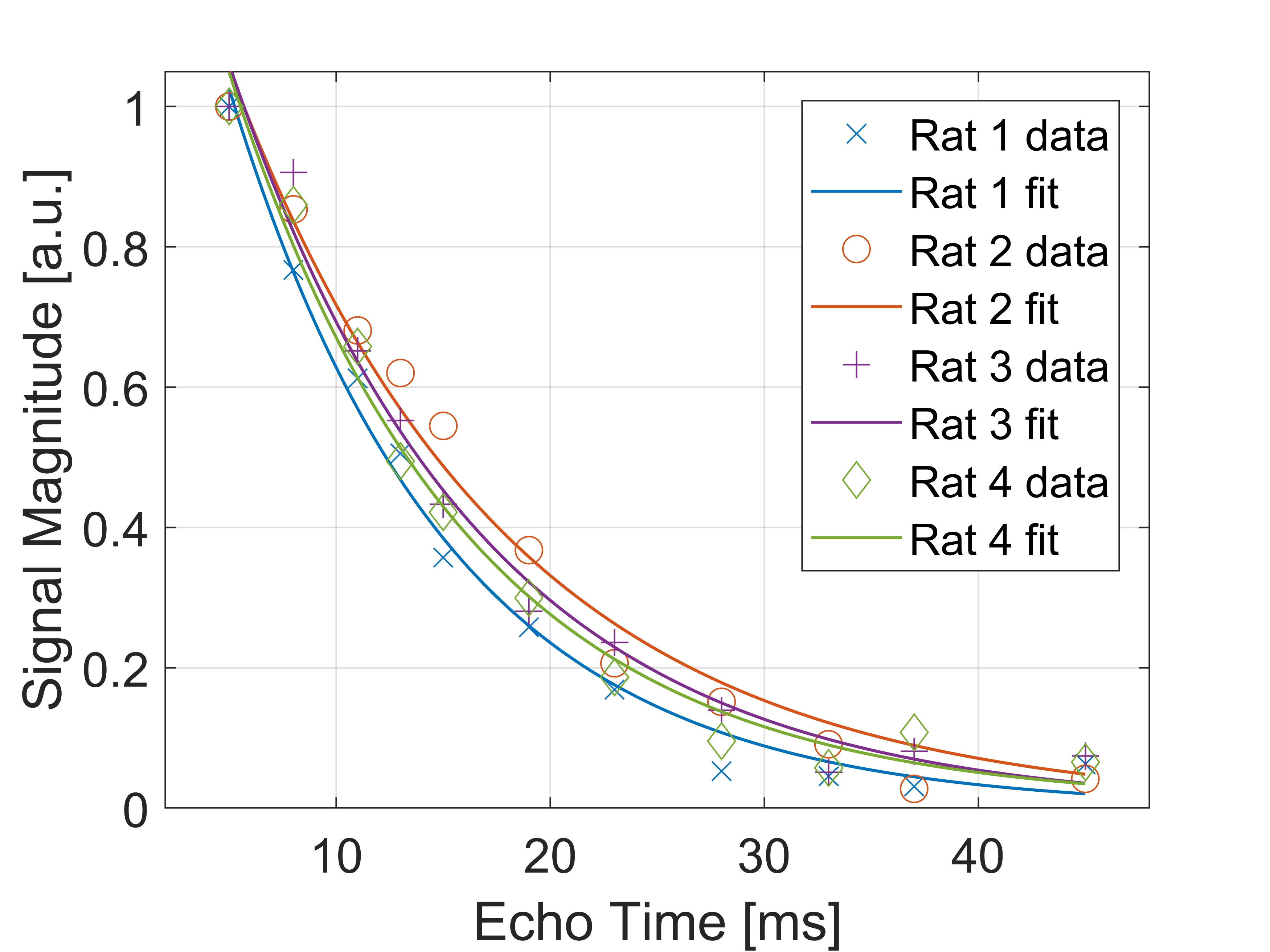

Supplement: S3 Fig — Transverse relaxation time of natural abundant deuterium in water (HDO) T2 = 11.4 ± 1.3 ms (mean and standard deviation), N = 4. (TIF) [file pone.0252935.s004.tif]
