# Supplementary material for: Feasibility of deuterium magnetic resonance spectroscopy of 3-O-Methylglucose at 7 Tesla
Source: PLoS One. 2021 Jun 7;16(6):e0252935. doi: 10.1371/journal.pone.0252935 (PMC8184010; doi:10.1371/journal.pone.0252935)
Supplement: S1 Table — All values are given as mean ± standard deviation. IR = inversion recovery, SE = spin echo, Glx = glutamate + glutamine; n. a. = not available, Water = HDO. (DOCX) [file pone.0252935.s005.docx]

**Feasibility of deuterium magnetic resonance spectroscopy of 3-O-Methylglucose at 7 Tesla**

Benedikt Hartmann, Max Müller, Lisa Seyler, Tobias Bäuerle, Tobias Wilferth, Nikolai Avdievitch, Loreen Ruhm, Anke Henning, Alexei Lesiv, Pavel Ivashkin, Michael Uder, Armin M. Nagel

S1 Table. Overview of relaxation times of deuterium.

| Reference | Substance | T_1_ (ms) | T_2_ (ms) | Field strength (T) | Description |
| --- | --- | --- | --- | --- | --- |
| This work | Water | 470 ± 1 | 458 ± 5 | 7 | Phantom with distilled water and additional D_2_O, 1 mol/l deuterium, IR, SE |
|  | Water | 270 ± 7 | 10.8 ± 0.4 | 7 | Phantom with 5 % Agarose, 20 mmol/l NaCl, IR, SE |
|  | D_2_O | 392 ± 1 | 375 ± 9 | 7 | Phantom, IR, SE, room temperature |
|  | OMG | 452 ± 2 | 406 ± 21 | 7 | Phantom containing 149 mmol/l OMG in distilled water, chemical shift 3.51 ppm, IR, SE |
|  | Water | 248 ± 7 | 11.4 ± 1.3 | 7 | In vivo, rat leg muscle, IR, SE, N = 4 |
| Ackerman 1987[1] | Water | 250 | n. a. | 8.5 | In vivo, rat liver, IR |
| Aguayo | Water | 446 | n. a. | 8.45 | In vitro, bacteria processed substances, IR |
| 1988[2] | Glucose | 290 | n. a. | 8.45 | In vitro, bacteria processed substances, IR |
| Assaf 1997[3] | Water | 329 ± 29 | 26.8 ± 0.8 | 8.46 | Excised rat brain, N = 4 |
|  |  | 354 ± 8 | 27 ± 1.7 | 11.75 | Excised rat brain, N = 2 |
|  |  | 272 ± 10 | 33.0 ± 0.8 | 8.46 | In vivo, rat brain, N = 3 |
| Bogin 2002[4] | Water | 186 ± 12 | n. a. | 4.7 | In vivo, mouse |
| Borle 1983[5] | Water | 502 | n. a. | 7 | Phantom, 25°C |
|  |  | 386 | n. a. | 7 | Phantom, 15°C |
| Brereton 1986[6] | Water | 228 ± 3 | n. a. | 4.7 | In vivo, mouse abdomen, 21 days after removal of D_2_O from drinking water, IR |
| Civan 1975[7] | Water | 143 ± 5.5 | n. a. | 1.24 | Excised frog muscle bathed in deuterium enriched (30-49%) Ringer’s solution, N = 4 |
| Cope 1969[8] | Water | 122 ± 2 | 8.5 ± 0.7 | 0.614 | Sample of rat muscle tissue, 28°C, 4 – 5 days of 2H enriched (50%) drinking water, N = 2 |
|  | Water | 131 ± 14 | 22 ± 12 | 0.614 | Sample of rat brain tissue, 25°C, 4 – 5 days of 2H enriched (50%) drinking water, N = 4 |
|  | Water | 453 ± 38 | 448 ± 4 | 0.614 | Phantom, IR, SE, 25°C, 10% D_2_O and 90% H_2_O |
|  | D_2_O | 407 ± 37 | n. a. | 0.614 | Phantom, IR, 25°C |
| De Feyter 2018[9] | Water | 346 ± 5 | 26 ± 1 (fast comp.)  351 ± 51 (slow comp.) | 4 | In vivo, human brain, bi-exponential T_2_, IR, SE, N = 2 |
|  |  | 320 ± 28 | 12 ± 2 (fast comp.)  31 ± 4 (slow comp.) | 11.7 | In vivo, rat brain, bi-exponential T_2_, IR, SE, N = 3 |
|  |  | 446 ± 7 | 372 ± 17 | 4 | Phantom with water |
|  |  | 462 ± 9 | 334 ± 21 | 11.7 | Phantom with water |
|  | Glucose | 67 ± 11 | 42 ± 1 | 4 | In vivo, human brain, D-Glucose-6,6-d_2_, IR, SE, N = 2 |
|  |  | 64 ± 2 | 32 ± 2 | 11.7 | In vivo, rat brain, IR, SE, N = 3 |
|  | Glx | 139 ± 11 | 44 ± 3 |  | In vivo, human brain, IR, SE, N = 2 |
|  |  | 146 ± 11 | 32 ± 5 | 11.7 | In vivo, rat brain, IR, SE, N = 3 |
| Eng 1990[10] | Water | 318 ± 8 | 72 ± 9 | 4.7 | In vivo, rabbit kidney, IR, SE, N = 7 (T1), N = 5 (T2) |
| Ewy 1988[11] | Water | 241 ± 9 | 9 ± 0.1 (fast comp.) 44 ± 5 (medium comp.)  370 ± 58 (slow comp.) | 4.7 | In vivo, cat head, IR, Hahn SE, N = 2 |
| Evelhoch 1989[12] | Water | 310 ± 30 | n. a. | 4.7 | In vivo, murine RIF-1 tumor, N = 3 |
| Hwang 1991[13] | Water | 300 ± 10 | n. a. | 4.7 | In vivo, murine RIF-1 tumor, N = 4 |
| Irving 1987[14] | Water | 228 ± 5 | n. a. | 4.7 | In vivo, mouse liver |
| Lu 2017[15] | Water | 360 ± 10 | n. a. | 16.4 | In vivo, rat brain, IR, N = 15 |
|  | Glucose | 50 ± 20 | n. a. | 16.4 | In vivo, rat brain, D-Glucose-6,6-d_2_, chemical shift 3.8 ppm, IR, N = 4 |
|  | Glx | 200 ± 50 | n. a. | 16.4 | In vivo, rat brain, chemical shift 2.4 ppm, IR, N = 3 |
|  | Water | 450 | n. a. | 16.4 | Phantom with glucose solution, IR |
|  | Glucose | 60 | n. a. | 16.4 | Phantom with glucose solution, IR |
| Mahar 2020[16] | Water | 490 | n. a. | 14.1 | In vitro cell sample, 25°C, IR |
| Woessner 1964[17] | D_2_O | 266  383  530  697 | n. a.  n. a.  n. a.  n. a. | 1.45 1.45 1.45 1.45 | Phantom, IR, 10°C  Phantom, IR, 20°C  Phantom, IR, 30°C  Phantom, IR, 40°C |

All values are given as mean ± standard deviation. IR = inversion recovery, SE = spin echo, Glx = glutamate + glutamine; n. a. = not available, Water = HDO

**References**

1. Ackerman JJ, Ewy CS, Becker NN, Shalwitz RA. Deuterium nuclear magnetic resonance measurements of blood flow and tissue perfusion employing 2H2O as a freely diffusible tracer. Proc Natl Acad Sci U S A. 1987;84(12):4099-102.

2. Aguayo JB, Gamcsik MP, Dick JD. High resolution deuterium NMR studies of bacterial metabolism. J Biol Chem. 1988;263(36):19552-7.

3. Assaf Y, Navon G, Cohen Y. In vivo observation of anisotropic motion of brain water using 2H double quantum filtered NMR spectroscopy. Magn Reson Med. 1997;37(2):197-203.

4. Bogin L, Margalit R, Ristau H, Mispelter J, Degani H. Parametric imaging of tumor perfusion with deuterium magnetic resonance imaging. Microvasc Res. 2002;64(1):104-15.

5. Borle F, Seelig J. Hydration of Escherichia coli lipids. Deuterium T1 relaxation time studies of phosphatidylglycerol, phosphatidylethanolamine and phosphatidylcholine. Biochim Biophys Acta. 1983;735(1):131-6.

6. Brereton IM, Irving MG, Field J, Doddrell DM. Preliminary studies on the potential of in vivo deuterium NMR spectroscopy. Biochemical and Biophysical Research Communications. 1986;137(1):579-84.

7. Civan MM, Shporer M. Pulsed nuclear magnetic resonance study of 17-O, 2-D, and 1-H of water in frog striated muscle. Biophysical Journal. 1975;15(4):299-306.

8. Cope FW. Nuclear Magnetic Resonance Evidence using D2O for Structured Water in Muscle and Brain. Biophysical Journal. 1969;9(3):303-19.

9. De Feyter HM, Behar KL, Corbin ZA, Fulbright RK, Brown PB, McIntyre S, et al. Deuterium metabolic imaging (DMI) for MRI-based 3D mapping of metabolism in vivo. Sci Adv. 2018;4(8):eaat7314.

10. Eng J, Berkowitz BA, Balaban RS. Renal distribution and metabolism of [2H9]choline. A 2H NMR and MRI study. NMR Biomed. 1990;3(4):173-7.

11. Ewy CS, Ackerman JJ, Balaban RS. Deuterium NMR cerebral imaging in situ. Magnetic Resonance in Medicine. 1988;8(1):35-44.

12. Evelhoch JL, McCoy CL, Giri BP. A method for direct in vivo measurement of drug concentrations from a single 2H NMR spectrum. Magnetic resonance in medicine. 1989;9(3):402-10.

13. Hwang YC, Kim SG, Evelhoch JL, Seyedsadr M, Ackerman JJ. Modulation of murine radiation-induced fibrosarcoma-1 tumor metabolism and blood flow in situ via glucose and mannitol administration monitored by 31P and 2H nuclear magnetic resonance spectroscopy. Cancer Res. 1991;51(12):3108-18.

14. Irving MG, Brereton IM, Field J, Doddrell DM. In vivo determination of body iron stores by natural-abundance deuterium magnetic resonance spectroscopy. Magn Reson Med. 1987;4(1):88-92.

15. Lu M, Zhu XH, Zhang Y, Mateescu G, Chen W. Quantitative assessment of brain glucose metabolic rates using in vivo deuterium magnetic resonance spectroscopy. J Cerebr Blood F Met. 2017;37(11):3518-30.

16. Mahar R, Donabedian PL, Merritt ME. HDO production from [(2)H7]glucose Quantitatively Identifies Warburg Metabolism. Sci Rep. 2020;10(1):8885.

17. Woessner DE. Molecular Reorientation in Liquids. Deuteron Quadrupole Relaxation in Liquid Deuterium Oxide and Perdeuterobenzene. The Journal of Chemical Physics. 1964;40(8):2341-8.
